# Supplementary material for: Studies of rice Hd1 haplotypes worldwide reveal adaptation of flowering time to different environments
Source: PLoS One. 2020 Sep 17;15(9):e0239028. doi: 10.1371/journal.pone.0239028 (PMC7498076; doi:10.1371/journal.pone.0239028)
Supplement: S3 Table — (DOCX) [file pone.0239028.s005.docx]

**S3 Table. ANOVA for flowering dates of *Hd1* haplotypes and the wild type.**

|  | Df | Sum Sq | Mean Sq | F value | Pr(>F) |  |
| --- | --- | --- | --- | --- | --- | --- |
| Haplotypes | 7 | 103334 | 14762 | 29.55 | <2e-16 | *** |
| Residuals | 2094 | 1046110 | 500 |  |  |  |
