# Supplementary material for: Studies of rice Hd1 haplotypes worldwide reveal adaptation of flowering time to different environments
Source: PLoS One. 2020 Sep 17;15(9):e0239028. doi: 10.1371/journal.pone.0239028 (PMC7498076; doi:10.1371/journal.pone.0239028)
Supplement: S4 Table — (DOCX) [file pone.0239028.s006.docx]

**S4 Table.** **Summary of statistical analysis of flowering dates of *Hd1* haplotypes and the wild type.**

| **Haplotype** | **N** | **Flowering days** | **SD** | **SE** | **CI** | **LSD group** | **Tukey HSD group** |
| --- | --- | --- | --- | --- | --- | --- | --- |
| WT | 1263 | 105.91 | 25.79 | 0.73 | 1.42 | a* | a* |
| 3 | 19 | 82.32 | 8.48 | 1.95 | 4.09 | c | b |
| 7 | 213 | 92.89 | 12.83 | 0.88 | 1.73 | b | b |
| 12 | 21 | 85.29 | 11.38 | 2.48 | 5.18 | bc | b |
| 13 | 552 | 92.37 | 16.88 | 0.72 | 1.41 | bc | b |
| 19 | 16 | 87.44 | 25.89 | 6.47 | 13.8 | bc | b |
| 20 | 15 | 88.67 | 7.35 | 1.9 | 4.07 | bc | b |
| 21 | 3 | 77.33 | 6.35 | 3.67 | 15.78 | c | b |

WT, wild type; SD, standard deviation; SE, standard error; CI, 95% confidence interval

* Different letters indicate significant difference at P<0.05.
