# Supplementary material for: Studies of rice Hd1 haplotypes worldwide reveal adaptation of flowering time to different environments
Source: PLoS One. 2020 Sep 17;15(9):e0239028. doi: 10.1371/journal.pone.0239028 (PMC7498076; doi:10.1371/journal.pone.0239028)
Supplement: S5 Table — Only types 7 and 13 are illustrated. Most are from 3K data and we added some accessions collected from Taiwan aboriginal villages. (DOCX) [file pone.0239028.s007.docx]

**S5 Table. Distribution of *japonica* and *indica* rice in traditional accessions from different countries.** Only Types 7 and 13 are illustrated. Most are from 3K data, and we added some accessions collected from Taiwan aboriginal villages.

|  | Type 7 | | | |  | Type 13 | | | |
| --- | --- | --- | --- | --- | --- | --- | --- | --- | --- |
|  | Total | Traditional | *japonica* | *indica* |  | Total | Traditional | *japonica* | *indica* |
| Bangladesh | 5 | 4 | -- | 4 |  | 78 | 35 | -- | 35 |
| Bhutan | 2 | 2 | 2 | -- |  | 2 | 2 | 1 | 1 |
| Cambodia | -- | -- | -- | -- |  | 1 | 1 | 1 | -- |
| China* | (120) | -- | (2) | (118) |  | (50) | -- | (9) | (41) |
| India | 40 | 3 | 1 | 2 |  | 108 | 16 | -- | 16 |
| Indonesia | 25 | 16 | 1 | 15 |  | 122 | 99 | 65 | 34 |
| Laos | 4 | 4 | -- | 4 |  | 2 | -- | -- | -- |
| Madagascar | 1 | -- | -- | -- |  | 25 | 8 | 3 | 5 |
| Malaysia | 8 | 2 | 2 | -- |  | 26 | 17 | 16 | 1 |
| Myanmar | 3 | 1 | -- | 1 |  | 3 | 0 | -- | -- |
| Pakistan | 1 | 1 | -- | 1 |  | 19 | 13 | -- | 13 |
| Philippines | 72 | 5 | -- | 5 |  | 105 | 62 | 47 | 15 |
| Sri Lanka | 5 | 1 | -- | 1 |  | 14 | 7 | 1 | 6 |
| Thailand | 7 | 1 | -- | 1 |  | 7 | 5 | 2 | 3 |
| Taiwan | 7 | 4 | -- | 4 |  | 3 | 3 | -- | 3 |
| Viet Nam | 10 | 3 | -- | 3 |  | 5 | 1 | -- | 1 |

* Because of no information on traditional/modern types for Chinese accessions, the total numbers are shown in brackets.
