# Supplementary material for: Studies of rice Hd1 haplotypes worldwide reveal adaptation of flowering time to different environments
Source: PLoS One. 2020 Sep 17;15(9):e0239028. doi: 10.1371/journal.pone.0239028 (PMC7498076; doi:10.1371/journal.pone.0239028)
Supplement: S9 Table — The Hd1 gene region and nearby ± 10-kb of traditional landraces were used for calculation. (DOCX) [file pone.0239028.s011.docx]

**Table S9. Selection parameter analysis of five *Hd1* types in several countries.** The *Hd1* gene region and nearby ± 10 kb of traditional landraces were used for calculation.

| Haplotype | Country | Subspecies | # of accessions | π | *θ*w | Tajima's *D* | Significance of *D* |
| --- | --- | --- | --- | --- | --- | --- | --- |
| Type 3 | Taiwan | *Indica* | 5 | 0.00151 | 0.00167 | -0.73046 | P > 0.10 |
| Type 7 | Bangladesh | *Indica* | 4 | 0.00065 | 0.00066 | -0.15923 | P > 0.10 |
| Type 7 | India | *Indica* and *japonica* | 3 | 0.00066 | 0.00066 | NA |  |
| Type 7 | India | *Indica* | 2 | 0.00054 | 0.00054 | NA |  |
| Type 7 | Indonesia | *Indica* and *japonica* | 16 | 0.00138 | 0.0027 | -2.12711 | **P < 0.01 |
| Type 7 | Indonesia | *Indica* | 15 | 0.00064 | 0.00096 | -1.4227 | P > 0.10 |
| Type 7 | Laos | *Indica* | 4 | 0.00044 | 0.00044 | -0.15777 | P > 0.10 |
| Type 7 | Malaysia | *Indica* and *japonica* | 2 | 0.00135 | 0.00135 | NA |  |
| Type 7 | Philippines | *Indica* | 5 | 0.00032 | 0.00032 | 0 | P > 0.10 |
| Type 7 | Taiwan | *Indica* | 3 | 0.00159 | 0.00159 | NA |  |
| Type 12 | Laos | *Japonica* | 10 | 0.00115 | 0.00179 | -1.75625 | * P < 0.05 |
| Type 13 | Indochina | *Indica* and *japonica* | 7 | 0.0007 | 0.00064 | 0.45159 | P > 0.10 |
| Type 13 | Bangladesh | *Indica* | 35 | 0.00294 | 0.00603 | -1.9545 | * P < 0.05 |
| Type 13 | India | *Indica* | 16 | 0.0032 | 0.00267 | 0.8686 | P > 0.10 |
| Type 13 | Indonesia | *Indica* and *japonica* | 99 | 0.0012 | 0.00547 | -2.65204 | *** P < 0.001 |
| Type 13 | Indonesia | *Indica* | 34 | 0.00079 | 0.0014 | -1.63627 | 0.10 > P > 0.05 |
| Type 13 | Indonesia | *Japonica* | 61 | 0.00137 | 0.00534 | -2.65105 | *** P < 0.001 |
| Type 13 | Malaysia | *Indica* and *japonica* | 17 | 0.0017 | 0.00295 | -1.82743 | * P < 0.05 |
| Type 13 | Malaysia | *Japonica* | 16 | 0.00176 | 0.00301 | -1.81371 | * P < 0.05 |
| Type 13 | Philippines | *Indica* and *japonica* | 62 | 0.00125 | 0.00432 | -2.52823 | *** P < 0.001 |
| Type 13 | Philippines | *Indica* | 15 | 0.00073 | 0.00082 | -0.47083 | P > 0.10 |
| Type 13 | Philippines | *Japonica* | 46 | 0.00137 | 0.00432 | -2.50592 | *** P < 0.001 |
| Type 19 | Taiwan | *Japonica* | 11 | 0.00205 | 0.00328 | -1.816 | * P < 0.05 |
