# Supplementary figures and images for: Studies of rice Hd1 haplotypes worldwide reveal adaptation of flowering time to different environments
Source: PLoS One. 2020 Sep 17;15(9):e0239028. doi: 10.1371/journal.pone.0239028 (PMC7498076; doi:10.1371/journal.pone.0239028)

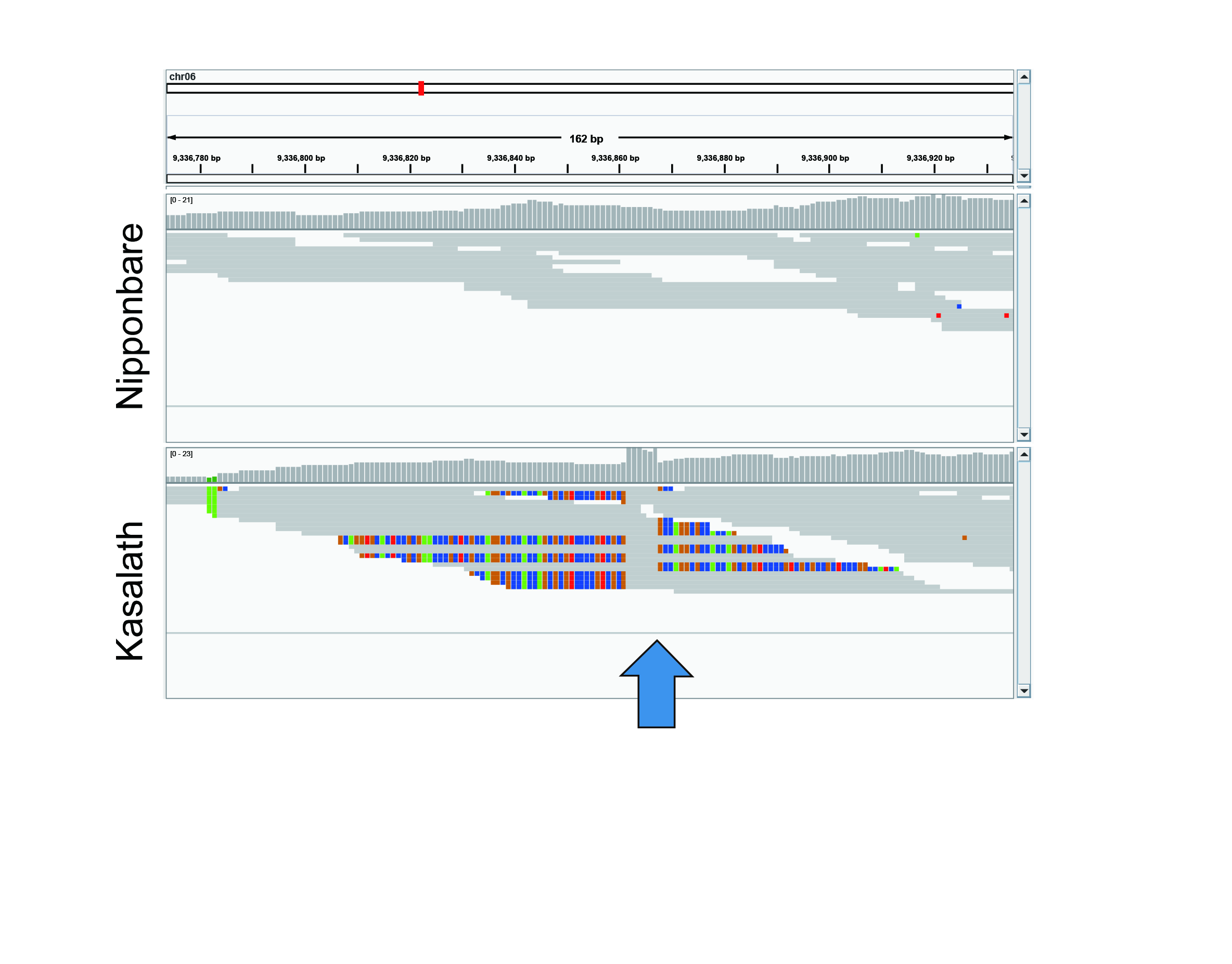

Supplement: S1 Fig — The Integrative Genomics Viewer view of aligned reads of a 162-bp first exon region of Hd1 gene is shown. The pair read sequencing was performed on an Illumina platform. Alignments toward the Nipponbare IRGSP 1.0 (top row) are represented as gray polygons and mismatched nucleotides as orange, blue, red and green bars. The second row is the alignment of Nipponbare Illumina reads and the third row the alignment of Kasalath reads. Blue arrow at bottom points to the 36-bp deletion region. (TIF) [file pone.0239028.s013.tif]

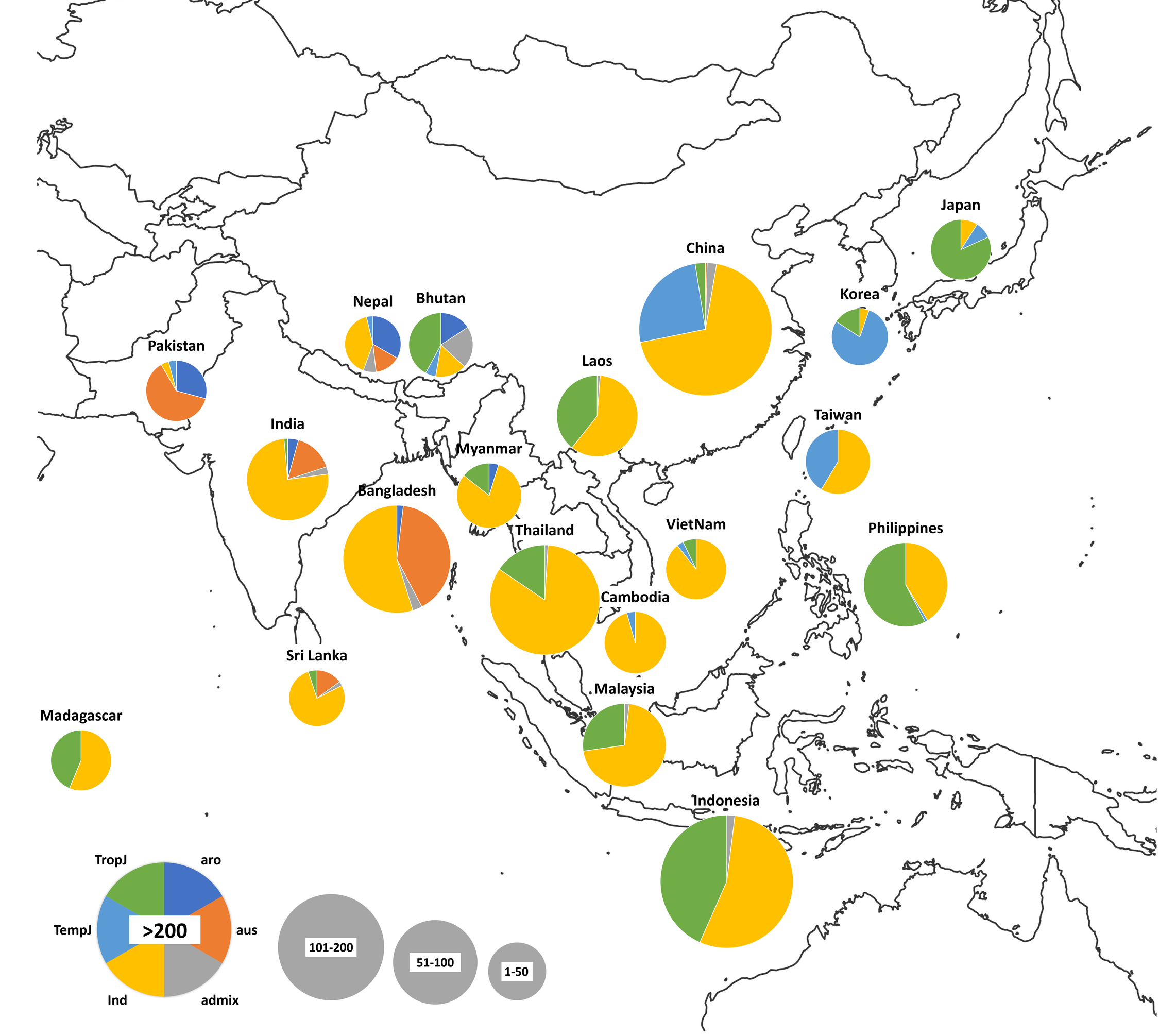

Supplement: S2 Fig — Colors indicate different subtypes and the pie size indicates the sample size. Only the traditional landraces in the 3K dataset are illustrated. Temperate japonica: light blue; tropical japonica: green; indica: yellow; aus: orange; aromatic: dark blue; admixture: grey. Because of no information about tradition/modern types for Chinese accessions, all accessions from China are used for illustration. The Asia map was downloaded from https://freevectormaps.com/world-maps/WRLD-EPS-01-0017. Republished from https://freevectormaps.com/world-maps/WRLD- EPS-01-0017 under a CC BY license, with permission from FreeVectorMaps.com, original copyright 2020. (TIF) [file pone.0239028.s014.tif]

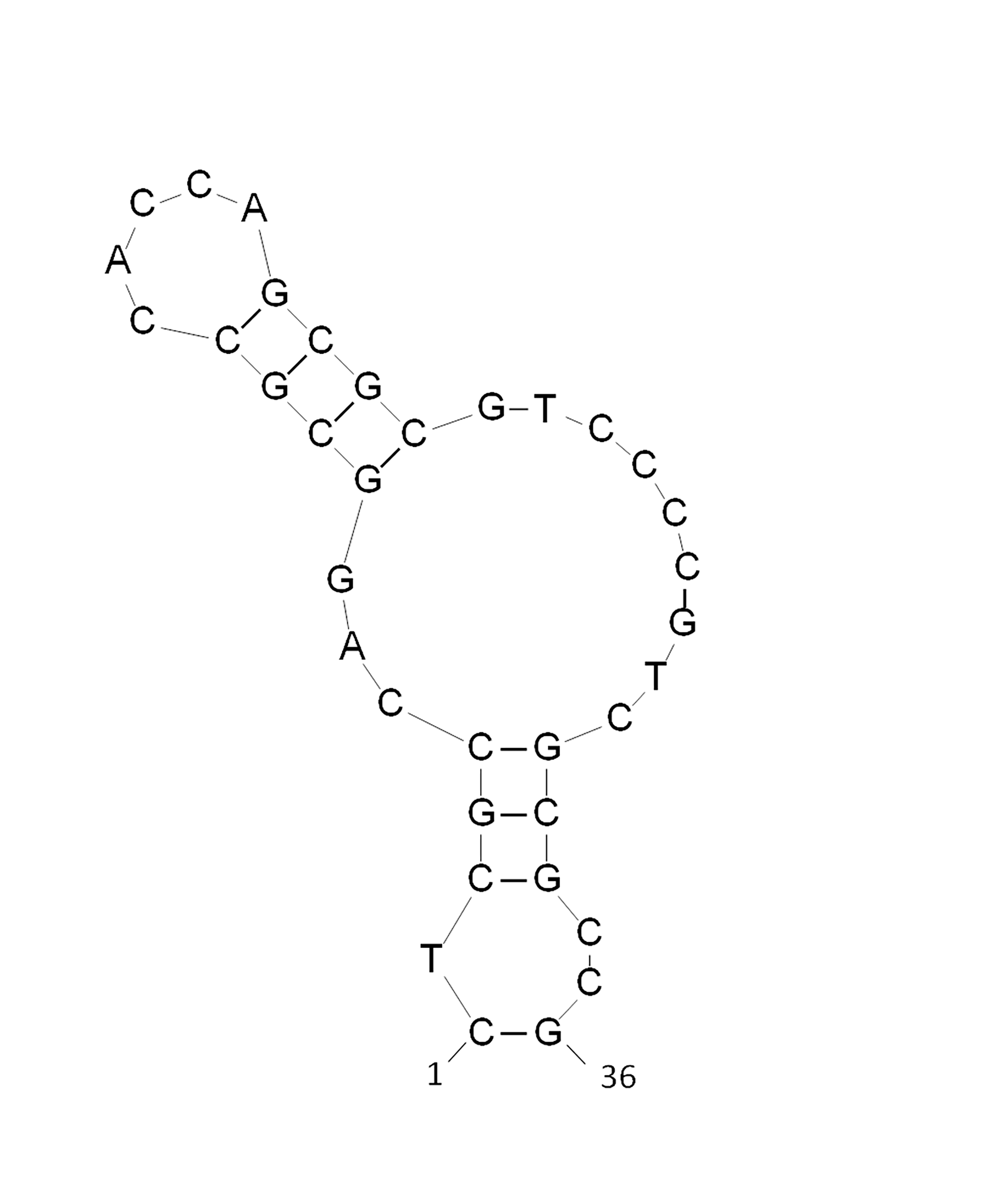

Supplement: S3 Fig — The mfold package at http://unafold.rna.albany.edu/?q=mfold was used. The free energy (dG) is -2.06 kcal mol-1. (TIF) [file pone.0239028.s015.tif]
