# Supplementary material for: Studies of rice Hd1 haplotypes worldwide reveal adaptation of flowering time to different environments
Source: PLoS One. 2020 Sep 17;15(9):e0239028. doi: 10.1371/journal.pone.0239028 (PMC7498076; doi:10.1371/journal.pone.0239028)
Supplement: Attachment — Submitted filename: Response to Reviewers.docx [file pone.0239028.s016.docx]

Reply to the comments

Thanks a lot to the academic editor and reviewers. We’ll reply to all points accordingly. The marked-up copy that highlights changes made to the original version is also provided.

Reply to the academic editor:

Firstly, I’d like to mention about one of the authors. Fu-Jin Wei was one of my former PhD students. He worked on the classification of the *Hd1* loss-of-function haplotype using the 3K data set before he graduated. He then moved to Tsukuba, Japan and worked on forestry genomics as postdoc for 2 years and just came back to Taiwan. He now works independently and hosts a private company for bioinformatics analysis (FourOneFour Studio). All the work he had done in *Hd1* gene analysis was carried out while he was my PhD student years ago. I’m very sorry we only put down his current address but not his previous affiliation while he worked on the *Hd1* gene.

Thus, for the Funding Statement: “The funder provided support in the form of salaries for author FJW years ago, but did not have any additional role in the study design, data collection and analysis, decision to publish, or preparation of the manuscript. The specific roles of this author is articulated in the “author contributions’ section.”

For Competing Interests Statement, “This does not alter our adherence to PLOS ONE policies on sharing data and materials.”

About the Figure 2 in the original submission and now the Figure 3 in the current version. We used the Asian map from <https://freevectormaps.com/world-maps/WRLD-EPS-01-0017>

They are free to use. Here is the link to the License Agreements <https://freevectormaps.com/license>

We added the information of the resource at the figure legend. We also thank to them in the Acknowledgement session. Thank you very much for the reminding.

Reply to reviewers’ comments

Reviewer 1

Thank you very much for the suggestions. I hereby answer accordingly.

Points 1 through 10:

Yes, we changed it according to the comments.

Points 11:

Table 3 was moved to S3 Table in the revision accordingly.

Point 12:

Thank you very much for the suggestion. We added it to the discussion and cited the reference accordingly. *Hd1* could have dual effect on rice production, i.e. involving in the rice development and controlling of flowering time.

Reviewer 2:

Thanks a lot for the critical comments and they certainly made improvements on this work. There are many major points so I reply one by one.

1.	The part related to Champa rice was indeed a different story and it was removed in this version.

2.	We performed allele network analysis as suggested. Because we only used the land races carrying types 7 and 13, a clear-cut network was shown (Fig. 2 in the current submission). We were able to show the area and types the mutations occurred and how they expanded by human migration. Together we demonstrated that introgressions and human activities played important roles for *hd1* diversification/adaptation.

3.	Thank you very much for pointing out how we detected 1.9 kb insertion in 3K data. I then double checked with my former PhD student FJW. He indeed wrote a python script 3 years ago and quickly scanned the whole data set followed by IGV confirmation. The method was updated and script provided as Supplementary Material.

4.	About the 36-bp insertion in the first exon. We performed the analysis and the results were shown in the revision. Because 36-bp insertion is very short, it is not easy to write a suitable script to scan the whole data set in large/fast scale. Thus, we used visualization identification as mentioned in the revision. Of the 1070 traditional landraces in 3K, all *indica* and tropical *japonica* accessions had this short insertion. Only few temperate *japonica* accessions were the same as Nipponbare, including 10 accessions from Korea, 6 from Japan, 4 from China, and 1 from Taiwan. For the 6 Japanese ones, 4 are real landraces (from NARO Genebank website), 1 is Nipponbare, and the other CX330 Yueguang. It took us a while to figure out this Yueguang should be Koshihikari as its Han characters (Kanji) pronounced as Yueguang in Chinese. It was picked up by CAAS and maybe even IRRI breeders do not know its real name is Koshihikari.

All 15 Asian wild rice tested also have this 36-bp fragment, however, 3 of them have identical or similar seq SNP with Nipponbare. We suggest the resource could be Japanese accession with 36-bp fragment. We do not have enough sequence information to check all though.

5. About which data was new and novel or which ones from previous work. Yes, we improve it accordingly. Thank you very much for pointing out.

Minor points

1. We added more discussion accordingly.
2. Yes, it was improved in the current session.
3. For the ~2500 accessions with seeds in IRRI germplasm, IRRI scientists performed a series of phenotyping analyses and data were available in SNP-SEEK, as mentioned in the ms. We downloaded these data and ran analysis. “Wild type” means the accessions without any LOF mutation and thus was used as outliers. Wild type accessions had the average heading date of 106 days, and the other types ranged from 77 to 93 days (S4 Table). The accession numbers used for WT, type 3, 7, 12, 13, 19, 20, 21 are 1263, 19, 213, 21, 552, 16, 16, and 3, respectively. We hope these data were enough to confirm they were LOF mutations.
4. There are 10 supplementary tables in the current version. For instances, we provided the SNPs information of *hd1* gene region of landraces carrying type 7 and 13 mutations, we also listed the accessions belong to each haplotype, etc. Because there are around 400 landraces consisting types 7 and 13 mutations, they were shown as Excel files in Supplementary tables.
