# Supplementary material for: Studies of rice Hd1 haplotypes worldwide reveal adaptation of flowering time to different environments
Source: PLoS One. 2020 Sep 17;15(9):e0239028. doi: 10.1371/journal.pone.0239028 (PMC7498076; doi:10.1371/journal.pone.0239028)
Supplement: Attachment — Submitted filename: Rebuttal_letter_0826.docx [file pone.0239028.s017.docx]

Rebuttal letter 0826

We hereby submit the 2^nd^ revision for our previous manuscript entitled “Studies of rice *Hd1* haplotypes worldwide reveal adaptation of flowering time to different environments”. Thanks a lot to the reviewers, and we modified the original manuscript accordingly.

Here are the points-to-points replies for the comments:

Reply to Reviewer #1:

1. Arabidopsis CO => CO

Yes, we changed them accordingly. Thank you very much.

2. typos or mistakes in pages 4, 14, 16 and 19

Yes, we changed them accordingly. Thank you very much.

Reply to Reviewer #2:

1. About the display of these subgroups (Fig 3). Yes, we added S2 Fig in the supplementary materials. It indeed improves the interpretation of Fig 3. Thank you very much.
2. About explanation of Tajima’s D results. Thank you very much for the reminding. We added a short paragraph for the interpretation accordingly.
